# Flavonoid Composition and Antitumor Activity of Bee Bread Collected in Northeast Portugal

**DOI:** 10.3390/molecules22020248

**Published:** 2017-02-07

**Authors:** Filipa Sobral, Ricardo C. Calhelha, Lillian Barros, Montserrat Dueñas, Andreia Tomás, Celestino Santos-Buelga, Miguel Vilas-Boas, Isabel C. F. R. Ferreira

**Affiliations:** 1 Mountain Research Centre (CIMO), ESA, Polytechnic Institute of Bragança, Campus de Santa Apolónia, 1172, 5300-253 Bragança, Portugal; filipa.as.sobral@gmail.com (F.S.); callelha@ipb.pt (R.C.C.); andreia-v-tomas@hotmail.com (A.T.); mvboas@ipb.pt (M.V.-B.); 2 Laboratory of Separation and Reaction Engineering—Laboratory of Catalysis and Materials (LSRE-LCM), Polytechnic Institute of Bragança, Campus de Santa Apolónia, 1134, 5301-857 Bragança, Portugal; 3 Grupo de Investigación en Polifenoles (GIP-USAL), Facultad de Farmacia, Universidad de Salamanca, Campus Miguel de Unamuno s/n, 37007 Salamanca, Spain; mduenas@usal.es (M.D.); csb@usal.es (C.S.-B.)

**Keywords:** bee bread, *Apis mellifera iberiensis*, phenolic compounds, HPLC-DAD-ESI/MS, cytotoxicity

## Abstract

Bee bread (BB) is a fermented mixture of plant pollen, honey, and bee saliva that worker bees use as food for larvae, and for young bees to produce royal jelly. In the present study, five BB samples, collected from *Apis mellifera iberiensis* hives located in different apiaries near Bragança, in the northeast region of Portugal, and one BB commercial sample were characterized by high-performance liquid chromatography coupled to a diode array detector and electrospray mass spectrometry (HPLC-DAD-ESI/MS) in terms of phenolic compounds, such as flavonoid glycoside derivatives. Furthermore, the samples were screened, using in vitro assays, against different human tumor cell lines, MCF-7 (breast adenocarcinoma), NCI-H460 (non-small cell lung cancer), HeLa (cervical carcinoma) and HepG2 (hepatocellular carcinoma), and also against non-tumor liver cells (porcine liver cells, PLP2). The main phenolic compounds found were flavonol derivatives, mainly quercetin, kaempferol, myricetin, isorhamnetin and herbacetrin glycoside derivatives. Thirty-two compounds were identified in the six BB samples, presenting BB1 and BB3 with the highest contents (6802 and 6480 µg/g extract, respectively) and the highest number of identified compounds. Two isorhamnetin glycoside derivatives, isrohamnetin-*O*-hexosyl-*O*-rutinoside and isorhamnetin-*O*-pentosyl-hexoside, were the most abundant compounds present in BB1; on the other hand, quercetin-3-*O*-rhamnoside was the most abundant flavonol in BB3. However, it was not possible to establish a correlation between the flavonoids and the observed low to moderate cytotoxicity (ranging from >400 to 68 µg/mL), in which HeLa and NCI-H460 cell lines were the most susceptible to the inhibition. To the authors’ knowledge, this is the first report characterizing glycosidic flavonoids in BB samples, contributing to the chemical knowledge of this less explored bee product.

## 1. Introduction

The nutritional requirements of honeybees, *Apis mellifera*, are met by the collection of pollen, nectar, and water. Nectar is the primary source of carbohydrates, while pollen provides proteins, lipids, vitamins and minerals [1]. Bee bread (BB) is a fermented mixture of plant pollen, honey, and bee saliva that worker bees use as food for larvae, and for young bees to produce royal jelly. Pollen collected by bees is mixed with a small amount of honey and saliva, and packed into the cells of the honeycomb where it undergoes a chemical change to form a product called bee bread [2]. This mixture undergoes different chemical processes due to the action of distinct enzymes from glandular secretions, microorganisms, moisture and temperature (35–36 °C chamber temperature offspring), allowing the transformation, improvement and preservation of the stored pollen, which is called bee bread after two weeks of initial storage [3,4].

Despite the role of BB as the main source of protein for the bees, its functional properties have been correlated, as well as its flavonoid content, with the BB’s floral origin [4]. In particular, BB has demonstrated in vitro antibacterial [5,6], antioxidant [3], and antitumor [2,7] properties. For the last activity, ethanolic extracts were screened against tumor cell lines (human glioblastoma cell line U87MG) and the normal human astroglia cell line SVGp12 (CRL-08621) using in vitro assays [2,7].

The BB composition varies according to the origin of the pollen but is mainly composed of water, proteins, carbohydrates, lipids, inorganic elements and various other minor components such as decanoic acid, gamma globulin, nucleic acids, vitamins B and C, pantothenic acid, biopterin, neopterin, acetylcholine, and reproductive hormones, among others [8]. 

The quality information available on the literature for bee bread remains limited, with few reports on the phenolic composition of this mixture. Some phenolic compounds were previously identified in BB samples from Poland, Russia, Latvia and Georgia [9,10]. Other reports on BB samples from Spain and Poland mentioned only total phenolics measured by the Folin-Ciocalteu colorimetric assay [2,4] and did not provide detailed characterization in terms of individual phenolic compounds. 

In the present study, five BB samples, collected from *Apis mellifera iberiensis* hives located in different apiaries near Bragança, in the northeast region of Portugal, and one sample of commercial BB were characterized by HPLC-DAD-ESI/MS in terms of their phenolic profile. Furthermore, the samples were screened against different human tumor cell lines, as well as against non-tumor liver cells. 

## 2. Results and Discussion

### 2.1. Chromatographic Profile of the BB

**Figure 1:**
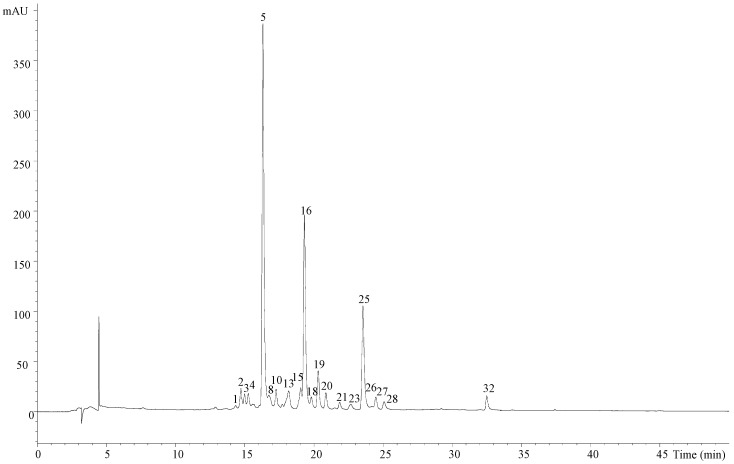
Individual phenolic compound profile of BB1 recorded at 370 nm. Peak numbering is the same as in Table 1 and Table 2.

The chromatographic profile of BB1 recorded at 370 nm can be observed in Figure 1; the peak characteristics, tentative identities and quantification of all the samples are presented in Table 1 and the quantification results are presented in Table 2. The main phenolic compounds found in bee bread were flavonol derivatives, mainly quercetin, kaempferol, myricetin, isorhamnetin and herbacetrin glycosides. The phenolic composition of bee bread has hardly been explored, only having been reported by a few authors [2,9,10], but using different analytical approaches. Tavdidishvili et al. [10] used HPLC-UV-Vis to study Georgian bee bread samples, reporting the presence of three flavonoids, naringin, rutin and quercetin. Isidorou et al. [9], using GC-MS, identified four phenolic acids (4-hydroxybenzoic, p-coumaric, ferulic and caffeic acids) and six flavonoids (chrysin, naringenin, kaempferol, isorhamnetin, apigenin and quercetin), whereas Markiewicz-Zukowska et al. [2], also using GC-MS, reported the presence of just two flavonoids, kaempferol and apigenin.

**Figure 2:**
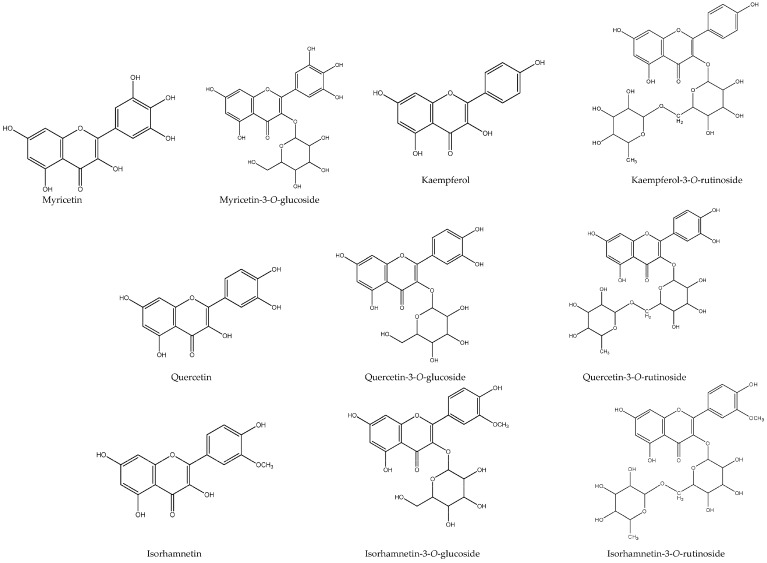
Flavonols’ chemical structures.

In our samples, up to 32 different flavonoids were detected (Table 1). Myricetin-3-*O*-glucoside (peak 7), quercetin-3-*O*-rutinoside (peak 10), kaempferol-3-*O*-rutinoside (peak 18), quercetin-3-*O*-glucoside (peak 19), isorhamnetin-3-*O*-rutinoside (peak 25) and isorhamnetin-3-*O*-glucoside (peak 28) were positively identified according to their retention, mass and UV-vis characteristics in comparison with commercial standards (Figure 2). Among them, peaks 10, 18 and 19 were found in all the studied samples, while compounds **7** (BB3), **25** (BB1) and **28** (BB1, BB3, BB5 and BBC) were only detected in some bee bread samples (Table 2). 

Peak 1 presented a pseudomolecular ion [M − H]*^−^* at *m*/*z* 625, releasing an MS^2^ fragment at *m*/*z* 317 ([M − H − 308]*^−^*, loss of a deoxyhexosyl-hexoside moiety), corresponding to myricetin. The presence of quercetin, kaempferol and isorhamnetin 3-*O*-rutinosides may point to peak 1 also a 3-*O*-rutinoside, and thus it was tentatively assigned as myricetin-3-*O*-rutinoside.

**Figure 3:**
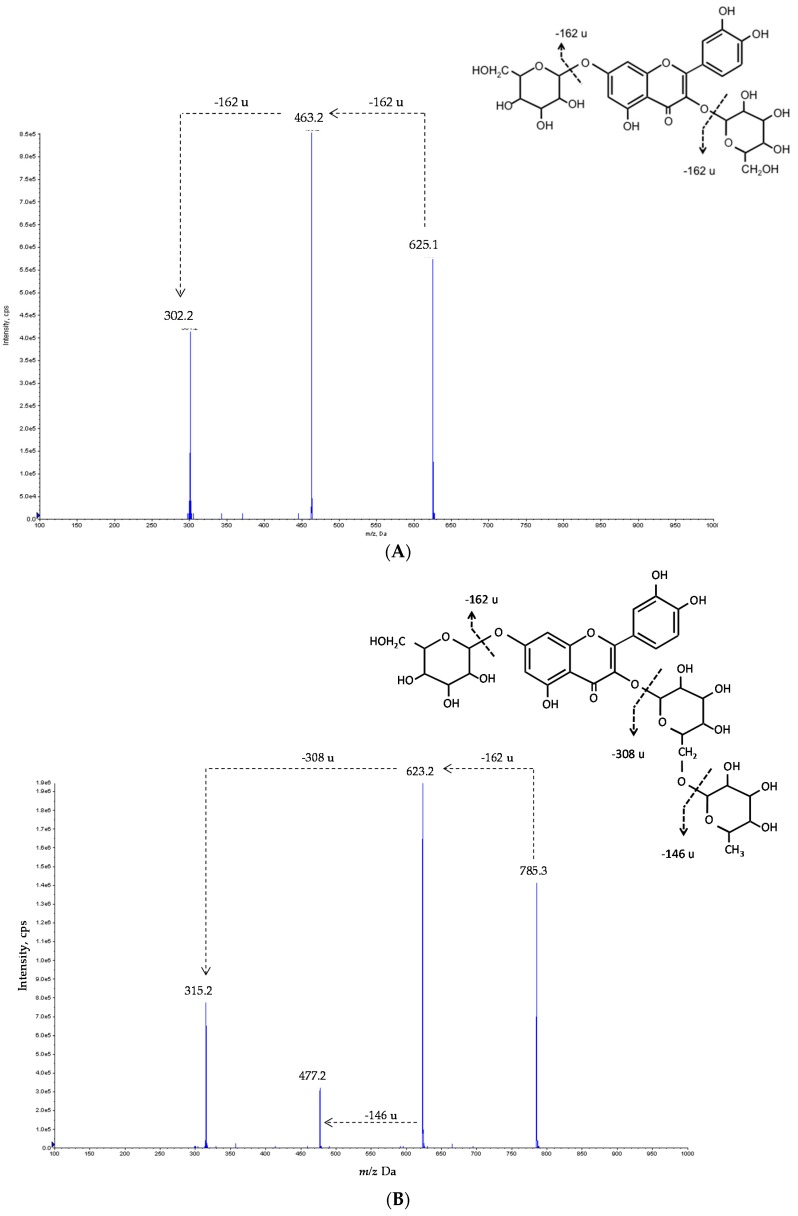
Fragmentation pattern of: (**A**) quercetin-3-*O*-glucoside-7-*O*-glucoside (possible identification for compound **4**) and (**B**) isorhamnetin-3-*O*-rutinoside-7-*O*-glucoside (possible identification for compound **5**).

Peaks 2, 4, 8, 9 and 26 were identified as quercetin derivatives owing to the product ion observed at *m*/*z* 301 and UV spectra (λ_max_ around 350–358 nm). Peaks 2 and 9 presented the same pseudomolecular ion [M − H]*^−^* at *m*/*z* 771. Peak 2’s MS^2^ fragments revealed the alternative loss of hexosyl (*m*/*z* at 609; −162 u) and deoxyhexosyl-hexoside (*m*/*z* at 463; −308 u) residues, indicating the location of each residue on different positions of the aglycone. Nevertheless, for peak 9 the observation of only one MS^2^ fragment suggested that the three sugars were linked together. For both peaks 2 and 9, no information about the identity of the sugar moieties and the location on the aglycone could be obtained, so the compounds were tentatively identified as quercetin-*O*-hexosyl-*O*-(deoxyhexosyl-hexoside) and quercetin-*O*-hexosyl-deoxyhexosyl-hexoside, respectively. Nevertheless, the positive identification of different rutinosides, including quercetin-3-*O*-rutinoside, in the analyzed samples may suggest a rutinose identity for the deoxyhexosyl-hexose residue present in peaks 2 and 9. The mass characteristics of peak 4 ([M − H]*^−^* at *m*/*z* 625) indicated that it corresponds to a quercetin derivative bearing two hexosyl residues. The observation of MS^2^ fragments at *m*/*z* 463 (−162 u) and 301 (−162 u) also indicated the alternative loss of each of the hexosyl moieties, respectively, pointing to their location on different positions of the aglycone. Thus, this compound was tentatively identified as quercetin-*O*-hexosyl-*O*-hexoside. Figure 3a exemplifies the fragmentation pattern of these types of compounds, and gives a tentative identification for peak 4. This compound was the majority flavonoid in the commercial sample and it was present in all the samples, with the exception of BB3 (Table 2).

Peaks 8 ([M − H]*^−^* at *m*/*z* 595) and 26 ([M − H]*^−^* at *m*/*z* 447) showed a similar fragmentation pattern as peak 9, only releasing one fragment at *m*/*z* 301, from the respective losses of pentosyl-hexoside (294 u) and deoxyhexose (146 u) moieties, and thus was tentatively assigned as a quercetin-*O*-pentosyl-hexoside and a quercetin-*O*-deoxyhexoside. This latter compound was detected in all the samples, being the majority flavonoid found in BB3 and BB5. Although MS analysis does not allow concluding about the nature and position of the substituting sugar, owing to its relatively high abundance in the analyzed samples, peak 26 can be speculated to correspond to quercitrin (i.e., quercetin-3-*O*-rhamnoside), a widespread flavonoid in plants.

Peaks 3, 12, 15, 27 and 30 were identified as kaempferol glycosides based on their UV spectra (λ_max_ around 348 nm) and the production of an MS^2^ fragment ion at *m*/*z* 285. Similarly, peaks 5, 16, 17, 20, 21 and 31 were identified as isorhamnetin (λ_max_ around 356 nm, MS^2^ fragment at *m*/*z* 315) glycosides. Tentative identities of these compounds were assigned based on their pseudomolecular ions using similar reasoning as for the quercetin derivatives. Thus, peaks 3 ([M − H]*^−^* at *m*/*z* 755) and 5 ([M − H]*^−^* at *m*/*z* 785) could correspond to kaempferol-*O*-hexosyl-*O*-(deoxyhexosyl-hexoside) and isorhamnetin-*O*-hexosyl-*O*-(deoxyhexosyl-hexoside), whereas peaks 15 and 17, with the same pseudomolecular ions as 3 and 5, could correspond to kaempferol-*O*-hexosyl-deoxyhexosyl-hexoside (peak 15) and isorhamnetin-*O*-hexosyl-deoxyhexosyl-hexoside (peak 17). As assumed for the quercetin derivatives, the positive identification of kaempferol and isorhamnetin 3-*O*-rutinoside allows us to speculate that peaks 3 and 5 correspond to kaempferol-*O*-hexosyl-*O*-rutinoside and isorhamnetin-*O*-hexosyl-*O*-rutinoside, respectively. Compound **5** was the majority flavonoid in sample BB1, and compound **15** in BB2 and BB4 samples. Figure 3b exemplifies the fragmentation pattern of these types of compounds, and gives a tentative identification for peak 5. Peaks 12 ([M − H]*^−^* at *m*/*z* 609), 27 ([M − H]*^−^* at *m*/*z* 563) and 30 ([M − H]*^−^* at *m*/*z* 431) could be assumed as a kaempferol-*O*-dihexoside, kaempferol-*O*-pentosyl-deoxyhexoside and kaempferol-*O*-deoxyhexoside, respectively. This latter might be supposed to be kaempferol-3-*O*-rhamnoside, based on the same considerations as for peak 26. 

Peaks 16, 20 and 21, mostly identified in sample BB1, showed the same pseudomolecular ion ([M − H]*^−^* at *m*/*z* 609), pointing out that they might correspond to different isorhamnetin-*O*-pentosyl-hexoside isomers. Peak 31 ([M − H]*^−^* at *m*/*z* 461), also bearing −146 u (loss of a deoxyhexosyl moiety), can be speculated to correspond to an isorhamnetin-*O*-deoxyhexoside, possibly isorhamnetin-3-*O*-rhamnoside.

Peaks 22 ([M − H]*^−^* at *m*/*z* 635) and 32 ([M − H]^−^ at *m*/*z* 519) possessed molecular weights 42 u higher than peaks 18 and 28, pointing to the existence of an additional acetyl residue, thus being tentatively identified as acetyl kaempferol-*O*-deoxyhexosyl-hexoside and acetyl isorhamnetin-*O*-hexoside, respectively. Similarly, peak 29 ([M − H]*^−^* at *m*/*z* 489) was tentatively identified as acetyl kaempferol-*O*-hexoside. 

Peaks 6, 11, 13, 14 and 23 were tentatively identified as methyl-herbacetin glycosides based on their UV spectra and the production of two MS^2^ fragments at *m*/*z* 315 and 300. This assignment was supported by the previous identification of similar compounds in bee pollen samples [11,12,13]. Compound identities were assigned based on their pseudomolecular ions, as methyl-herbacetin-*O*-deoxyhexosyl-hexoside (peak 13), methyl-herbacetin-*O*-hexoside (peak 23), methyl-herbacetin-*O*-hexosyl-deoxyhexosyl-hexoside (peak 11) and two methyl-herbacetin-*O*-dihexoside isomers (peaks 6 and 14). Peaks 13 and 23 can be speculated to correspond respectively to methyl-herbacetin-3-*O*-rutinoside and methyl-herbacetin-3-*O*-glucoside, taking into account the presence in the samples of equivalent glycosides derived from other flavonols. With the exception of peak 11, which was only found in sample BB5, the rest of the methyl-herbacetin derivatives were detected in most of the analyzed samples (Table 2).

Finally, peak 24 ([M − H]*^−^* at *m*/*z* 477), only detected in sample BB3, showed a similar fragmentation pattern as the methyl-herbacetin derivatives, releasing in this case two MS^2^ fragment ions at *m*/*z* 331 and 315, and was tentatively identified as a methylmyricetin derivative, possibly laricitrin (i.e., 3′-*O*-methylmyricetin). According to its pseudomolecular ion, it would be a laricitrin-*O*-deoxyhexoside, which can also be speculated to correspond to laricitrin-3-*O*-rhamnoside owing to the detection of other rhamnosides in the sample (peaks 26 and 30). To the authors' knowledge, this is the first report on the presence of flavonol glycoside derivatives in bee bread.

### 2.2. Antitumoral Activity of the BB Samples

The BB samples showed some toxicity against the four human tumor cell lines were used: MCF-7 (breast adenocarcinoma), NCI-H460 (non-small cell lung cancer), HeLa (cervical carcinoma) and HepG2 (hepatocellular carcinoma). However, at the tested concentrations, the samples inhibited less than 50% of the growth of the tumor cells, the results being expressed in terms of GI_25_ values (sample concentration providing 25% of growth inhibition) (Table 3). BBC was selective for the HeLa cell line, while BB3 inhibited the growth of all the tested human tumor cell lines, being the only one that was able to inhibit HepG2 growth. Besides the mentioned data, BB1 and mostly BB2 were also active against MCF-7, BB4 and BB5 against NCI-H460, and BB1, BB5 and principally BB4 against HeLa. It should be highlighted that up to 400 µg/mL, none of the BB samples showed toxicity for normal cells (non-tumor porcine liver primary cells).

Despite the reports on the antitumor properties of different phenolic compounds, including flavonoids [14], it was not possible to establish a positive correlation between the concentration of flavonoids in each sample and the corresponding cytotoxicity. Therefore, these properties could be attributed to specific individual flavonoids, synergism/antagonism dynamics in the samples and, moreover, the presence of other compounds rather than flavonoids.

## 3. Materials and Methods

### 3.1. Samples Collection 

The samples of BB were collected in 2012 from *Apis mellifera* iberiensis hives located in different apiaries near Bragança, in the northeast region of Portugal; specifically located in Bragança (BB1), Montesinho (BB2), Rio de Onor (BB3), Vinhais (BB4), and Castrelos (BB5). For extraction, the frames next to the bee brood were removed from the hives, freeze and the wax crushed mechanically to collect the bee bread. An additional commercial sample (BBC), collected from *Apis dorsata* on the Himalayan region, was courtesy of Bee Healthy Farms (Springfield, MO, USA).

Once extracted, the samples were lyophilized (FreeZone 4.5 model 7750031, Labconco, Kansas City, MO, USA), reduced to a fine dried powder (20 mesh), mixed to obtain homogenous samples and stored in a desiccator, protected from light, until further analysis. 

### 3.2. Standards and Reagents

HPLC-grade acetonitrile was obtained from Merck KgaA (Darmstadt, Germany). Formic and acetic acids were purchased from Fisher Scientific (Waltham, MA USA). The phenolic compound standards (myricetin-3-*O*-glucoside, quercetin-3-*O*-rutinoside, kaempferol-3-*O*-rutinoside, quercetin 3-*O*-glucoside, isorhamnetin-3-*O*-rutinoside and isorhamnetin-3-*O*-glucoside) were from Extrasynthese (Genay, France). The cell lines HeLa, HEPG2, NCI-H460 and MCF-7 were purchased from Deutshe Sammlung von Mikroorganismen and Zellkulturen Gmbit (DSMZ, Braunschweig, Germany). Dulbecco’s modified Eagle’s medium (DMEM), hank’s balanced salt solution (HBSS), fetal bovine serum (FBS), l-glutamine, trypsin-EDTA, penicillin/streptomycin solution (100 U/mL and 100 mg/mL, respectively) were purchased from Gibco Invitrogen Life Technologies (Carlsbad, CA, USA). Water was treated in a Milli-Q water purification system (TGI Pure Water Systems, Greenville, SC, USA).

### 3.3. Extracts Preparation

A methanol:water (80:20, *v*/*v*) extract was obtained from the lyophilized material. Each sample (1 g) was extracted twice by stirring (25 °C at 150 rpm) with 30 mL of methanol:water (80:20, *v*/*v*) for 1 h and subsequently filtered through a Whatman No. 4 paper. The combined methanol:water extracts were evaporated at 40 °C (rotary evaporator Büchi R-210, Flawil, Switzerland) to remove the methanol and further frozen and lyophilized. 

### 3.4. Characterization of the Extracts by HPLC-DAD-ESI/MS 

The phenolic compounds were analysed using a Hewlett-Packard 1100 chromatograph (Hewlett-Packard 1100, Agilent Technologies, Santa Clara, CA, USA) with a quaternary pump and a diode array detector (DAD) coupled to an HP Chem Station (rev. A.05.04) data-processing station. A Waters Spherisorb S3 ODS-2 C18, 3 μm (4.6 mm × 150 mm) column thermostatted at 35 °C was used and 10 µL of each sample was injected. The solvents used were: (A) 0.1% formic acid in water, (B) acetonitrile. The elution gradient established was 15% B for 5 min, 15% B to 20% B over 5 min, 20%–25% B over 10 min, 25%–35% B over 10 min, 35%–50% B for 10 min, and re-equilibration of the column, using a flow rate of 0.5 mL/min. Double online detection was carried out in the DAD using 280 nm and 370 nm as preferred wavelengths and in a mass spectrometer (MS) connected to HPLC system via the DAD cell outlet.

MS detection was performed in an API 3200 Qtrap (Applied Biosystems, Darmstadt, Germany) equipped with an ESI source and a triple quadrupole-ion trap mass analyser that was controlled by the Analyst 5.1 software. Zero grade air served as the nebulizer gas (30 psi) and turbo gas for solvent drying (400 °C, 40 psi). Nitrogen served as the curtain (20 psi) and collision gas (medium). The quadrupoles were set at unit resolution. The ion spray voltage was set at −4500 V in the negative mode. The MS detector was programmed for recording in two consecutive modes: Enhanced MS (EMS) and enhanced product ion (EPI) analysis. EMS was employed to show full scan spectra, so as to obtain an overview of all of the ions in sample. Settings used were: declustering potential (DP) −450 V, entrance potential (EP) −6 V, collision energy (CE) −10 V. EPI mode was performed in order to obtain the fragmentation pattern of the parent ion(s) in the previous scan using the following parameters: DP −50 V, EP −6 V, CE −25 V, and collision energy spread (CES) 0 V. Spectra were recorded in negative ion mode between *m*/*z* 100 and 1000.

The phenolic compounds present in the samples were characterised according to their UV and mass spectra and retention times compared with standards, when available. For the quantitative analysis of phenolic compounds, a fiev-level calibration curve was obtained by injection of known concentrations (2.5–100 µg/mL) of different standards compounds: isorahmetin-3-*O*-glucoside (*y* = 218.26*x* − 0.98; *R*^2^ = 0.999); isorahmetin-3-*O*-rutinoside (*y* = 284.12*x* + 67.055; *R*^2^ = 0.999); kaempferol-3-*O*-glucoside (*y* = 288.55*x* − 4.05; *R*^2^ = 1); kaempferol-3-*O*-rutinoside (*y* = 182.94*x* + 96.644; *R*^2^ = 1); quercetin-3-*O*-glucoside (*y* = 236.33*x* + 70.006; *R*^2^ = 0.999) and quercetin-3-*O*-rutinoside (*y* = 280.87*x* + 0.37373; *R*^2^ = 1). The results were expressed in µg per g of extract.

### 3.5. Evaluation of In Vitro Cytotoxic Properties of the Extracts

The BB extracts were dissolved in water at 8 mg/mL and then submitted to further dilutions from 400 to 1.56 µg/mL. Four human tumor cell lines were used: MCF-7 (breast adenocarcinoma), NCI-H460 (non-small cell lung cancer), HeLa (cervical carcinoma) and HepG2 (hepatocellular carcinoma). Cells were routinely maintained as adherent cell cultures in RPMI-1640 medium containing 10% heat-inactivated FBS and 2 mM glutamine (MCF-7, NCI-H460 HeLa and HepG2 cells), at 37 °C, in a humidified air incubator containing 5% CO_2_. Each cell line was plated at an appropriate density (7.5 × 10^3^ cells/well for MCF-7 and NCI-H460 or 1.0 × 10^4^ cells/well for HeLa and HepG2) in 96-well plates. Sulforhodamine B assay was performed according to a procedure previously described by the authors [15].

For evaluation of the cytotoxicity in non-tumor cells, a cell culture was prepared from a freshly harvested porcine liver obtained from a local slaughterhouse, according to a procedure established by the authors [16]; it was designed as PLP2. Cultivation of the cells was continued with direct monitoring every two to three days using a phase contrast microscope. Before confluence was reached, cells were subcultured and plated in 96-well plates at a density of 1.0 × 10^4^ cells/well, and commercial in DMEM medium with 10% FBS, 100 U/mL penicillin and 100 µg/mL streptomycin. Ellipticine was used as positive control and all the results were expressed in GI25 values (concentration that inhibited 25% of the net cell growth). 

### 3.6. Statistical Analysis

Data on phenolic compounds quantification were expressed as mean ± standard deviation. Differences in the phenolic compounds levels among each of the assayed bee bread samples were analyzed through one-way analysis of variance (ANOVA). The fulfilment of the one-way ANOVA requirements, specifically the normal distribution of the residuals and the homogeneity of variance, was tested by means of the Shapiro Wilk’s and the Levene’s tests, respectively. All dependent variables were compared using Tukey’s honestly significant difference (HSD) or Tamhane’s T2 multiple comparison tests, when homoscedasticity was verified or not, respectively. For all compounds detected in only two bee bread samples, statistically significant differences among means were classified according to *t*-student test. All statistical tests were performed at a 5% significance level using SPSS software, version 23.0 (IBM Corp., Armonk, NY, USA). 

## 4. Conclusions

Overall, bee bread is a very recent bee product and at this stage it can be collected and consumed as a food supplement named “bee bread”. To the authors’ knowledge, this is the first report characterizing glycosidic flavonoids in bee bread samples, contributing to the chemical knowledge of this less explored bee product. Thirty-two flavonol glycoside derivatives, such as quercetin, kaempferol, myricetin, isorhamnetin and herbacetrin derivatives, were identified in the six samples of bee bread. BB1 and BB3 were the bee bread samples that presented the highest content and the highest number of identified compounds (19 compounds). Two isorhamnetin glycoside derivatives, isrohamnetin-*O*-hexosyl-*O*-rutinoside and isorhamnetin-*O*-pentosyl-hexoside, were the most abundant compounds present in BB1; on the other hand, quercetin-3-*O*-rhamnoside was the most abundant flavonol in BB3. BB samples showed moderate antitumor activity; however, none of the BB samples have shown toxicity for normal cells. 

## Figures and Tables

**Table 1:** Retention time (Rt), wavelengths of maximum absorption in the visible region (λ_max_), mass spectral data and identification of phenolic compounds in bee bread samples.

Peak	Rt (min)	λ_max_ (nm)	Molecular ion [M − H]^−^ (*m*/*z*)	MS^2^ (*m*/*z*)	Identification
1	14.3	358	625	317 (100)	Myricetin-3-*O*-rutinoside
2	14.7	350	771	609 (100), 463 (9), 301 (23)	Quercetin-*O*-hexosyl-*O*-rutinoside
3	14.9	346	755	593 (100), 447 (21), 285 (34)	Kaempferol-*O*-hexosyl-*O*-rutinoside
4	15.3	350	625	463 (100), 301 (48)	Quercetin-*O*-hexosyl-*O*-hexoside
5	16.3	350	785	623 (100), 477 (16), 315 (30)	Isorhamnetin-*O*-hexosyl-*O*-rutinoside
6	16.3	350	639	315 (18), 300 (21)	Methyl herbacetrin-*O*-dihexoside
7	16.5	354	479	317 (100)	Myricetin-3-*O*-glucoside
8	16.7	358	595	301 (100)	Quercetin-*O*-pentosyl-hexoside
9	16.8	356	771	301 (100)	Quercetin-*O*-hexosyl-rutinoside
10	17.3	358	609	301 (100)	Quercetin 3-*O*-rutinoside
11	17.4	350	785	315 (32), 300 (20)	Methyl herbacetrin-*O*-hexosyl-rutinoside
12	17.7	348	609	285 (100)	Kaempferol-*O*-dihexoside
13	18.2	352	623	315 (36), 300 (22)	Methyl herbacetrin-3-*O*-rutinoside
14	18.4	350	639	315 (29), 300 (14)	Methyl herbacetrin-*O*-dihexoside
15	19.0	350	755	285 (100)	Kaempferol-*O*-hexosyl-rutinoside
16	19.3	356	609	315 (100)	Isorhamnetin-*O*-pentosyl-hexoside
17	19.6	354	785	315 (100)	Isorhamnetin-*O*-hexosyl-rutinoside
18	19.8	348	593	285 (100)	Kaempferol-3-*O*-rutinoside
19	20.3	356	463	301 (100)	Quercetin-3-*O*-glucoside
20	20.9	354	609	315 (100)	Isorhamnetin-*O*-pentosyl-hexoside
21	21.9	356	609	315 (100)	Isorhamnetin-*O*-pentosyl-hexoside
22	22.6	350	635	285 (100)	Acetyl kaempferol-*O*-deoxyhexosyl-hexoside
23	22.7	346	477	315 (50), 300 (33)	Methyl herbacetrin-3-*O*-glucoside
24	23.2	356	477	331 (19), 315 (32)	Laricitrin-3-*O*-rhamnoside
25	23.5	356	623	315 (100)	Isorhamnetin-3-*O*-rutinoside
26	24.2	350	447	301 (100)	Quercetin-3-*O*-rhamnoside
27	24.5	348	563	285 (100)	Kaempferol-*O*-pentosyl-deoxyhexoside
28	25.1	356	477	315 (100)	Isorhamnetin-3*-O*-glucoside
29	26.7	350	489	285 (100)	Acetyl kaempferol-*O*-hexoside
30	28.8	346	431	285 (100)	Kaempferol-3-*O*-rhamnoside
31	29.9	350	461	315 (100)	Isorhamnetin-3-*O*-rhamnoside
32	32.5	356	519	315 (100)	Acetyl isorhamnetin-*O*-hexoside

**Table 2:** Quantification of the phenolic compounds (µg/g of extract) present in the bee bread samples.

	BB1	BB2	BB3	BB4	BB5	BBC	Normal Distribution ^1^	Homoscedasticity ^2^	Differences among Means ^3^
Myricetin-3-*O*-rutinoside	41 ± 3c	nd	322 ± 7a	nd	38 ± 4c	118 ± 4b	0.002	0.719	<0.001
Quercetin-*O*-hexosyl-*O*-rutinoside	156 ± 8	nd	nd	nd	nd	nd	-	-	-
Kaempferol- *O*-hexosyl-*O*-rutinoside	69 ± 1	nd	nd	nd	nd	nd	-	-	-
Quercetin-*O*-hexosyl-*O*-hexoside	129 ± 5c	211 ± 6b	nd	127 ± 4c	74 ± 3d	1580 ± 31a	<0.001	0.089	<0.001
Isorhamnetin-*O*-hexosyl-*O*-rutinoside	2615 ± 54	nd	nd	nd	nd	nd	-	-	-
Methyl herbacetrin-*O*-dihexoside	nd	622 ± 25a	70 ± 3d	460 ± 3b	192 ± 1c	nd	0.046	0.084	<0.001
Myricetin-3-*O*-glucoside	nd	nd	36 ± 2	nd	nd	nd	-	-	-
Quercetin-*O*-pentosyl-hexoside	100 ± 5	nd	nd	nd	nd	139 ± 1	0.023	0.148	<0.001
Quercetin-*O*-hexosyl-rutinoside	nd	314 ± 6b	106 ± 9c	367 ± 1a	nd	nd	0.006	0.290	<0.001
Quercetin 3-*O*-rutinoside	158 ± 3c	88 ± 5e	312 ± 5b	105 ± 6d	91 ± 2e	377 ± 7a	0.001	0.688	<0.001
Methyl herbacetrin-*O*-hexosyl-rutinoside	nd	nd	nd	nd	217 ± 1	nd	-	-	-
Kaempferol-*O*-dihexoside	nd	91 ± 6c	246 ± 8b	83 ± 4c	108 ± 1c	1167 ± 30a	<0.001	0.094	<0.001
Methyl herbacetrin-3-*O*-rutinoside	186 ± 25c	nd	71 ± 1d	435 ± 5a	225 ± 7b	nd	0.037	0.119	<0.001
Methyl herbacetrin-*O*-dihexoside	nd	nd	39 ± 3d	164 ± 13b	268 ± 11a	105 ± 5c	0.152	0.424	<0.001
Kaempferol-*O*-hexosyl-rutinoside	212 ± 10d	3597 ± 69b	403 ± 1c	3755 ± 46a	nd	130 ± 2e	<0.001	0.095	<0.001
Isorhamnetin-*O*-pentosyl-hexoside	1448 ± 37	nd	nd	nd	nd	nd	-	-	-
Isorhamnetin-*O*-hexosyl-rutinoside	nd	103 ± 1	nd	43 ± 3	nd	nd	0.009	0.208	<0.001
Kaempferol-3-*O*-rutinoside	62 ± 7de	94 ± 21d	355 ± 10c	56 ± 4e	815 ± 16b	1627 ± 32a	<0.001	0.300	<0.001
Quercetin-3-*O*-glucoside	248 ± 5a	52 ± 8e	236 ± 1b	53 ± 3e	177 ± 3c	72 ± 1d	0.001	0.249	<0.001
Isorhamnetin-*O*-pentosyl-hexoside	94 ± 8	nd	nd	nd	nd	nd	-	-	-
Isorhamnetin-*O*-pentosyl-hexoside	30 ± 2	nd	47 ± 3	nd	nd	nd	0.081	0.743	<0.001
Acetyl kaempferol-*O*-deoxyhexosyl-hexoside	nd	nd	20 ± 1	nd	11 ± 1	nd	0.037	0.639	<0.001
Methyl herbacetrin-3-*O*-glucoside	tr	32 ± 4d	53 ± 6c	224 ± 10a	138 ± 17b	nd	0.033	0.383	<0.001
Laricitrin-3-*O*-rhamnoside	nd	nd	125 ± 5	nd	nd	nd	-	-	-
Isorhamnetin-3-*O*-rutinoside	836 ± 35	nd	nd	nd	nd	nd	-	-	-
Quercetin-3-*O*-rhamnoside	tr	280 ± 22c	3029 ± 72a	168 ± 19d	2001 ± 17b	190 ± 7d	0.001	0.236	<0.001
Kaempferol-*O*-pentosyl-deoxyhexoside	82 ± 6	nd	nd	nd	nd	nd	-	-	-
Isorhamnetin-3*-O*-glucoside	140 ± 1b	nd	199 ± 1a	nd	118 ± 4c	64 ± 2d	0.103	0.269	<0.001
Acetyl kaempferol-*O*-hexoside	nd	nd	nd	nd	nd	22 ± 3	-	-	-
Kaempferol-3-*O*-rhamnoside	nd	nd	141 ± 12	nd	29 ± 10	nd	0.038	0.747	<0.001
Isorhamnetin-3-*O*-rhamnoside	nd	73 ± 10c	670 ± 44a	nd	232 ± 14b	nd	0.022	0.234	<0.001
Acetyl isorhamnetin-*O*-hexoside	197 ± 12	nd	nd	nd	nd	nd	-	-	-
Total flavonoids	6802 ± 204a	5557 ± 179d	6480 ± 128b	6040 ± 76c	4733 ± 106e	5593 ± 118d	0.417	0.804	<0.001

nd: not detected; tr: traces. ^1^ Normal distribution of the residuals was evaluated using Shapiro-Wilk test (*p* > 0.05 indicates normal distribution). ^2^ Homoscedasticity among bread formulations was tested by Levene’s test: homoscedasticity, *p* > 0.05; heteroscedasticity, *p* < 0.05. ^3^ *p* < 0.05 indicates that the mean value of the corresponding phenolic compound of at least one formulation differs from the others, allowing us to perform multiple comparison tests (Tukey’s HSD for homoscedastic distributions, Tamhane’s T2 for heteroscedastic distributions); for all phenolic compounds detected only in two bee bread samples, differences among means were compared by Student’s *t*-test. For each bee bread sample, means within a line with different letters differ significantly (*p* < 0.05).

**Table 3:** Cytotoxic activity (GI_25_ values, µg/mL) of the bee bread (BB) samples.

	Human Tumor Cell Lines				Non-Tumor Porcine Liver Cells
	MCF-7	NCI-H460	HeLa	HepG2	PLP2
BB1	186 ± 6a	>400	345 ± 13a	>400	>400
BB2	84 ± 3c	>400	>400	>400	>400
BB3	164 ± 4b	253 ± 10a	225 ± 12bc	67 ± 1	>400
BB4	>400	85 ± 5b	209 ± 21c	>400	>400
BB5	>400	68 ± 8b	276 ± 18b	>400	>400
BBC	>400	>400	366 ± 7a	>400	>400
Ellipticine	0.45 ± 0.02	0.74 ± 0.01	0.55 ± 0.03	1.61 ± 0.07	1.06 ± 0.02

GI_25_ values: sample concentration providing 25% of growth inhibition in human tumor cell lines or in liver primary culture PLP2. In each column different letters mean significant statistical differences (*p* < 0.05).
